# Editorial: The impact of exercise intervention with the internet and wearable devices on mental health

**DOI:** 10.3389/fpsyg.2024.1348725

**Published:** 2024-01-11

**Authors:** Meng Ding, Xiao Hou, Xiaosheng Dong

**Affiliations:** ^1^School of Physical Education, Shandong Normal University, Jinan, China; ^2^School of Sport science, Beijing Sport University, Beijing, China; ^3^Centre for Health Management and Policy Research, School of Public Health, Cheeloo College of Medicine, Shandong University, Jinan, China

**Keywords:** exercise, internet, wearable device, mental health, tele-medicine

With the development of Internet technology, remote exercise instruction has been widely developed. However, there is a paucity of research on the effects of tele-coaching on participants' psychological status and quality of life. This column is set up to make such research accessible to interested readers as soon as possible. Since its inception, this column has published four major papers on college students, elite athletes and cancer patients.

Wang et al. compares the differential effects of different training modalities based on tele-coaching high intensity interval training and aerobic combined with resistance training on the physical and psychological wellbeing of college students in a randomized, parallel-controlled study. The authors randomized the participants into a HIIT group and an AR group. Participants' psychological wellbeing indexes, physical fitness indexes, and body composition indexes were measured before and after the experimental intervention. After the 8 weeks of intervention, it was found that for college students' physical fitness levels and body composition, both tele-coaching high intensity interval training and aerobic combined with resistance training showed some improvements. It is worth noting that high intensity interval training is more effective in the improvement of aerobic endurance. As for psychological health, tele-coaching-based HIIT may produce more significant positive effects than combined exercise. The findings suggest that remote coaching HIIT is both time- and space-independent as well as strengthening, better highlighting the benefits of remote coaching exercise compared to traditional exercise.

Yunchao et al. assesses the use of virtual simulation in sports decision-making training and also discusses the advantages of virtual simulation in improving decision-making skills, as well as the limitations and challenges it may face. Authors searched several databases of academic literature and analyzed the filtered findings in order to identify the actual use or effectiveness of virtual simulation technology in sports decision-making. The included articles were coded, including identifying and documenting key information about the study design, study participant characteristics, virtual reality task setup, experimental interventions, and study outcomes. Research has shown that virtual simulation technology has been widely used in a diverse range of sports scenarios, especially in the simulation of sports decision-making tasks. The technology can effectively track and record the decisions made by athletes during the game for subsequent analysis. Under specific conditions, virtual reality environments help to improve perceptual and cognitive abilities and provide significant assistance in training for sports decision-making. Therefore, the future should be devoted to the scientific design of virtual simulation environments for sports decision-making. The findings of the study provide an important foundation for more effective use of virtual simulation to enhance sports decision-making.

Cui et al. delves into whether people's physical activity and sleep were affected during the COVID-19 pandemic. In the study, attention was not only paid to the impact itself, but also focused on the influence of the measurement methods and the characteristics of the study population on the results. The authors used multiple database systems to retrieve relevant researches. The authors systematically evaluated the 11 articles included in the meta-analysis by collecting and analyzing the characteristics and data from the studies therein that were relevant to the topic, and estimating combined effect sizes in studies. It has been found that the COVID-19 pandemic significantly affects moderate to vigorous physical activity (MVPA) time, with respondents reducing or changing their level of exercise during this period. The pandemic also had a significant impact on sleep duration and sleep quality, with people changing their sleep habits or quality during this period. Additionally, for healthy adults, overall physical activity levels did not change significantly despite the reduction in MVPA time. Outcomes of MVPA time and sleep duration are susceptible to being affected by the method in which they are measured, and additionally, individuals vary in their sleep behaviors depending on the level of physical activity in different populations. Therefore, measurement methods should be considered when studying physical activity and population differences could be noted when studying sleep behavior. The findings of this study are an important aid in developing responses related to physical and mental health relevant responses in possible future epidemic scenarios.

Zhu et al. examines the use of an exercise rehabilitation app in breast cancer patients undergoing Radio/Chemotherapy and its effects on their quality of life, sleep, and psychology. The authors used a mixed-methods study to more fully analyze the app's improvement of side effects in breast cancer patients during Radio/Chemotherapy by combining both quantitative and qualitative data. The authors asked the participants to use the exercise rehabilitation app to perform aerobic exercises and rehabilitation exercises during Radio/Chemotherapy. The authors assessed the exercise rehabilitation app's appropriateness by examining participant satisfaction, app usage, and the effect on life quality, sleep quality, as well as levels of anxiety and depression. The study revealed favorable outcomes in terms of the frequency of exercise rehabilitation app usage and participant evaluations during Radio/Chemotherapy. The findings suggest that employing the exercise rehabilitation app could be effective in mitigating the adverse effects of treatment side impacts on life quality, sleep quality, as well as levels of anxiety and depression in breast cancer patients undergoing Radio/Chemotherapy. The results of this study provide important insights for clinicians, breast cancer patients, and patients' families regarding exercise rehabilitation interventions after breast cancer surgery.

## Author contributions

MD: Writing—original draft. XH: Writing—review & editing. XD: Writing—review & editing.

